# Blockchain-enabled architecture for lead acid battery circularity

**DOI:** 10.1038/s41598-024-67404-z

**Published:** 2024-07-16

**Authors:** Deepika Choudhary, Kuldip Singh Sangwan, Arpit Singh

**Affiliations:** Department of Mechanical Engineering, Birla Institute of Technology and Science Pilani, Pilani, Rajasthan 333031 India

**Keywords:** Lead acid battery, Material circularity, Blockchain, Supply chain, Traceability, Hazardous and critical materials, Climate sciences, Environmental sciences, Environmental social sciences, Natural hazards

## Abstract

Widespread use of lead acid batteries (LABs) is resulting in the generation of million tons of battery waste, globally. LAB waste contains critical and hazardous materials, which have detrimental effects on the environment and human health. In recent times, recycling of the LABs has become efficient but the collection of batteries in developing countries is not efficient, which led to the non-professional treatment and recycling of these batteries in the informal sector. This paper proposes a blockchain-enabled architecture for LAB circularity, which ensures authentic, traceable and transparent system for collection and treatment of batteries. The stakeholders—battery manufacturers, distributors, retailers, users, and validators (governments, domain experts, third party experts, etc.)—are integrated in the circular loop through a blockchain network. A mobile application user interface is provided to all the stakeholders for the ease of adoption. The batteries manufactured and supplied in a geographical region as well as the recycled materials at the battery end-of-life are traced authentically. This architecture is expected to be useful for the battery manufacturers to improve their extended producer responsibility and support responsible consumption and production.

## Introduction

The rising aspirations of the emerging nations and the increasing reliance on technology are contributing to the higher generation of E-waste. Battery waste, a significant contributor to E-waste, is rapidly increasing due to its multipurpose uses in automobiles, home appliances, and industries^1^. LAB is one of the oldest rechargeable battery technologies used for the energy storage for over a century^2^. The high usage and popularity of LABs can be attributed to their low cost, voltage stability, easy maintenance, and wide range of applications^3^. In many developing countries, electric power backup is a major requirement as the grid electricity supply is erratic and not available 24 hours a day. This forces the households and small commercial establishments to have an inverter-based (battery-based) power backup. These inverters for the electric power backup use lead acid batteries (LABs). This means the batteries are distributed across the small villages and hamlets making the reverse supply chain complex. LABs are generally used for motive and stationary applications. According to the international lead association (ILA), around 60% of the LABs are used in automotive sector. Stationary batteries are generally used for backup power supply like home appliances, data centers & telecommunication, forklifts, cranes, marines, etc. The global market for the LABs was 79.9 billion USD in 2021 and is forecasted to reach 115.1 billion USD during 2022–2030 with a compound annual growth rate of 2.52%. The growth of LABs across the globe is expected to continue^4^. The large population and the rising aspirations of the emerging and developing nations will keep the battery market and thus battery waste increasing.

LAB mainly consists of 60–68% of lead, 0.28% of tin, 0.03% of calcium, 17–22% of electrolyte (hydrogel) containing 38.5% diluted sulfuric acid in solid state, 4–6% silicon dioxide, and 4–12% casing material (acrylonitrile butadiene styrene or polypropylene) by percentage weight^5^. The non-professional disposal of hazardous LAB materials causes severe environmental impairments. Lead contamination affects kidney, cardiovascular, and nerve system. Sulfuric acid is corrosive and causes direct tissue damage to animals and plants, soil acidification, nutrient leaching, disruption of mycorrhiza symbiosis, etc.^1,6^. The spent LABs can be recycled to get secondary metallic and non-metallic materials which are either critical (scarce) or hazardous. Nearly 86% of the lead used in the LAB manufacturing comes from recycling and about 60% of the recycled lead is used to fulfill world’s lead requirement^7,8^. Recycling of LABs creates jobs and also reduces exposure to hazardous materials, promoting planet health^9^. Recycled lead requires less energy and hence generates lesser environmental impacts as compared to virgin lead production. Sulfuric acid can be recycled or treated to be used in industries for detergent, fertilizer, textile, and glass manufacturing^10^. These economic, environmental and social benefits motivate the recycling of LABs.

Many countries have established facilities for safe treatment, scientific recycling, and disposal of LABs. In the European Union, the LAB supply chains are fully operative in closed loop and economically sustainable. The LAB recycling efficiency for the EU and USA is reported as high as 99%^11^, this number for Australia is 97%^10^. In fact, recycling of LABs is one of the prodigious recycling achievements. Approximately 90% of the total available batteries in the Australian countries are recycled and remaining 10% LABs could not be collected for authentic recycling. However, a study of India shows that about 90% of spent LABs go to informal recycling centers, wherein batteries are treated and recycled in non-professional manner and the remaining waste and toxins are dumped causing ground, air, and water pollution^12^. It has been observed that, despite having close to 100% recycling efficiency, the spent LABs are collected back in a small percentage for scientific recycling, at least in developing countries, and half of the spent LABs recycling culminates in the informal sectors leading to non-profession disposal^13,14^. A white paper by world economic forum (WEF) and global battery alliance (GBA) estimates around 10,000–30,000 informal battery recycling sites globally. Africa, India, Indonesia, and Bangladesh are among the regions devastated by informal recycling^8^. Therefore, an authentic and traceable solution is required to prevent informal recycling, unauthorized landfilling, and illegal export of LABs.

Lack of regulations, poor implementation of regulations, complex supply chain, conventional untraceable identity of end user, lack of authenticity in management and collection mechanisms are the major causes of informal recycling in many countries. The manner of recycling by the informal sector wherein materials like acid and plastics are dumped into water bodies or landfills without treatment are the major causes of the non-professional treatment and disposal of the LABs^14^. Today, blockchain technology is used for the proper implementation of government regulation at the operational level in the financial sector. This can be extended to the proper implementation of policies, regulations, and guidelines related to LABs collection, recycling and disposal.

There is a gap between the generation & collection, and collection & recycling of LABs despite having good recycling efficiency. The first and the biggest hurdle is the authentic collection of the batteries. The traceability of the products along the supply chain can help to improve the collection. Also, the extended producer responsibility (EPR) regime aiming at target-based recovery systems for waste collection and recycling, based on production and sales statistics, can be highly effective and easy to implement for the manufacturers if the products can be traced throughout their life cycle. Therefore, newer methods or architectures are required for the effective management of collection and recycling to ensure the circularity of the LABs. Tracing out the battery and its materials can be a judicious step for the effective management and accounting of the LAB waste. But due to complexity of the supply chain, it’s difficult to trace out a LAB throughout its life span using traditional methods. Many recent advances in industrial engineering like digitalization of supply chains, use of industry 4.0 concepts^15,16^, and circular economy^17^ have been adopted for the ease of tracing the products/parts along the supply chain. However, these techniques/methodologies do not improve authenticity and transparency in the system. Recently, blockchain technology has gained the attention of researchers and businesses to enhance traceability, transparency, and authenticity in the field of supply chain and circular economy^18^. Blockchain technologies can provide the potent solutions to resolve the issue of tracing the product from cradle-to-grave and ensure the authenticity of collected parts to prevent or reduce fraudulent activities^19,20^. This paper proposes a blockchain-enabled architecture which can trace a LAB and its materials, with authenticity and transparency, until the material is recirculated or repurposed.

## Literature survey

### Lead acid battery supply chain and circular economy

Recycling has become essential to practice responsible consumption and manage waste to minimize the burden on the planet earth. Sasikumar and Haq^21^ designed a multi echelon, multi product closed loop supply chain network for automotive SLI (starting, lighting and ignite) batteries, integrated with third party reverse logistics for achieving reverse logistics cost efficiencies and delivery schedules. But there was no authentic mechanism defined for collection methods. Langarudi et al.^22^ conducted a study on reverse logistics frameworks for automotive LABs consisting of collection, remanufacturing, repair, recycling, and disposal to minimize the cost and CO_2_ using mathematical modeling. However, this study also does not provide authentic methods of collection. Tracing out the material throughout the value chain is a complex task. The materials supply chains have complex connections among its actors and hence are difficult to trace back and forth. Panza et al.^16^ proposed the idea of a digital material passport for EV batteries, which enhances the circularity of products and ease of maintenance, thus enabling sustainable product management. But it does not provide a transparent system, which may lead to malpractices in recycling at the end-of-life. Also, it does not talk about the product end-of-life activities. Scur et al.^23^ proposed a triple bottom line based concept for the LAB close loop supply chain. The proposed study presents an approach to achieve sustainability in LAB supply chains. However, the study lacks authenticity in the recycling processes and accounting of recovered materials.

### Blockchain-enabled product or material tracing throughout the supply chain

The concept of blockchain was developed as a research project in 1991, but first application as a cryptocurrency Bitcoin, was developed by an anonymous person “Satoshi Nakamoto”^24^. He developed a trustless, peer to peer network for electronic transactions using proof-of-work consensus. Eventually, the cryptocurrencies like Bitcoin, Litecoin, Ripple, Dogecoin, etc., started replacing the trust-based systems with the trustless finance system using cryptography^25^. As the result of further development, blockchain technology has been classified based on the applications– financial & non-financial; and based on the accessibility—permissioned/private, non-permissioned/public, consortium, hybrid, etc.^26^. Blockchain technology has also been adopted for supply chain traceability^27,28^. However, many open-ended questions still exist related to blockchain viability in real case supply chains with respect to scalability, response time, reliability of consensus protocols^29^ & security issues such as phishing attacks, stolen keys, routing attacks, Sybil attacks, etc.^30^. Boubaker et al.^31^ presented a hypothesis-based road map to test blockchain feasibility, and tested hypotheses related to scalability and response time. But the proposed study does not talk about traceability methods related to real industrial and social problems. The study by Grassi et al.^19^ aims to enhance the information exchange between vendors and buyers in digital supply chains using blockchain technology to reduce inventories, provide highly customized products, and preserve confidential information by introducing intermediate entities. However, this study does not involve the backward supply chain, and cannot take care of non-professional treatment, recycling and disposal of products. Some researchers in the extant literature have used blockchain technology for the e-waste management. Khan and Ahmad^32^ attempted tracking, tracing, and recycling of E-waste in smart cities by using IoT and blockchain technology based methods. Objective of the proposed work was to ensure the recycling of E-waste without compromising the privacy of stakeholders, and used smart waste bins with sensors and an image detection model for smart city environments. The study carried out a viability analysis of blockchain architecture. However, this framework is for the segregation of E-waste at product level. Moreover, the proposed sensor based bins are expensive and the framework is based on the assumption that citizens will dump the E-waste in these bins. Poongodi et al.^33^ suggested an Ethereum based e-waste tracing and proposed an incentive mechanism to channelize the e-waste to the government-authorised agencies for the effective environment friendly disposal, which helps in reducing the disposal by unorganised sectors. Farizi et al.^34^ deployed hyperledger fabric architecture for e-waste management along with the Geographic Information System (GIS) and Google Map technology for ease of locating the collection sites. They also analysed the transaction time and data storage methods for supporting circular economy. Kholiya et al.^35^ developed smart contracts using blockchain technology for secure and transparent e-waste management framework. Ambre et al.^36^ developed a hyperledger based e-waste management using smart contracts to personalise the e-waste pickups for small-scale businesses and households and used tokens for compensation. Santhuja and Anbarasu^37^ developed a blockchain and IoT based framework for e-waste collection and management. The study depicts the smart bin concept for e-waste identification using RFID tags and IoT sensors for e-waste monitoring in a city. Sahoo et al.^38^ suggested global e-waste management solution using a proof-of-concept using solidity on the ethereum platform and performed experimental evaluations to demonstrate its feasibility in terms of execution gas cost. Dasaklis et al.^39^ proposed a distributed trustless and secure framework for e-waste (mobile phone) reverse logistic activities using ethereum blockchain.

However, in general, the above-mentioned e-waste management techniques using blockchain technology lacks material level accounting. Also, most of these papers are about reverse logistic activities for authentic and transparent recycling and do not record and account the recovered material for circularity and better resource management. For example, in case of LAB, if material level accounting is not recorded authentically then there are chances that cheaper but harmful material like used acid can be disposed off in non-professional manner to avoid cost associated with acid transportation and treatment. This paper proposes a blockchain-based architecture for authentic, transparent and traceable system for collection and treatment of LABs not only to track the LABs but also to track its recovered material to enhance and support EPR.

### Research gaps and objectives of the study

Concerned authorities and researchers are trying to find effective methods for LAB collection and recycling to improve recycling efficiency for extracting maximum secondary material out of spent batteries. It is observed that research to digitalize the forward supply chain of LABs has been attempted to integrate the forward supply chain stakeholders. There is hardly any blockchain-enabled study in the literature on LABs or even E-waste, which integrates the manufacturers, distributors, retailers, recyclers, government authorities, and users to account for the materials used in the products and their recycling.

Major objective of the proposed study is to develop a blockchain-enabled solution for enhancing the traceability, transparency and authenticity of LAB’s critical and hazardous materials to ensure the circularity of critical materials and safe treatment, and scientific recycling and disposal of hazardous materials.

## Materials and methods

**Figure 1:**
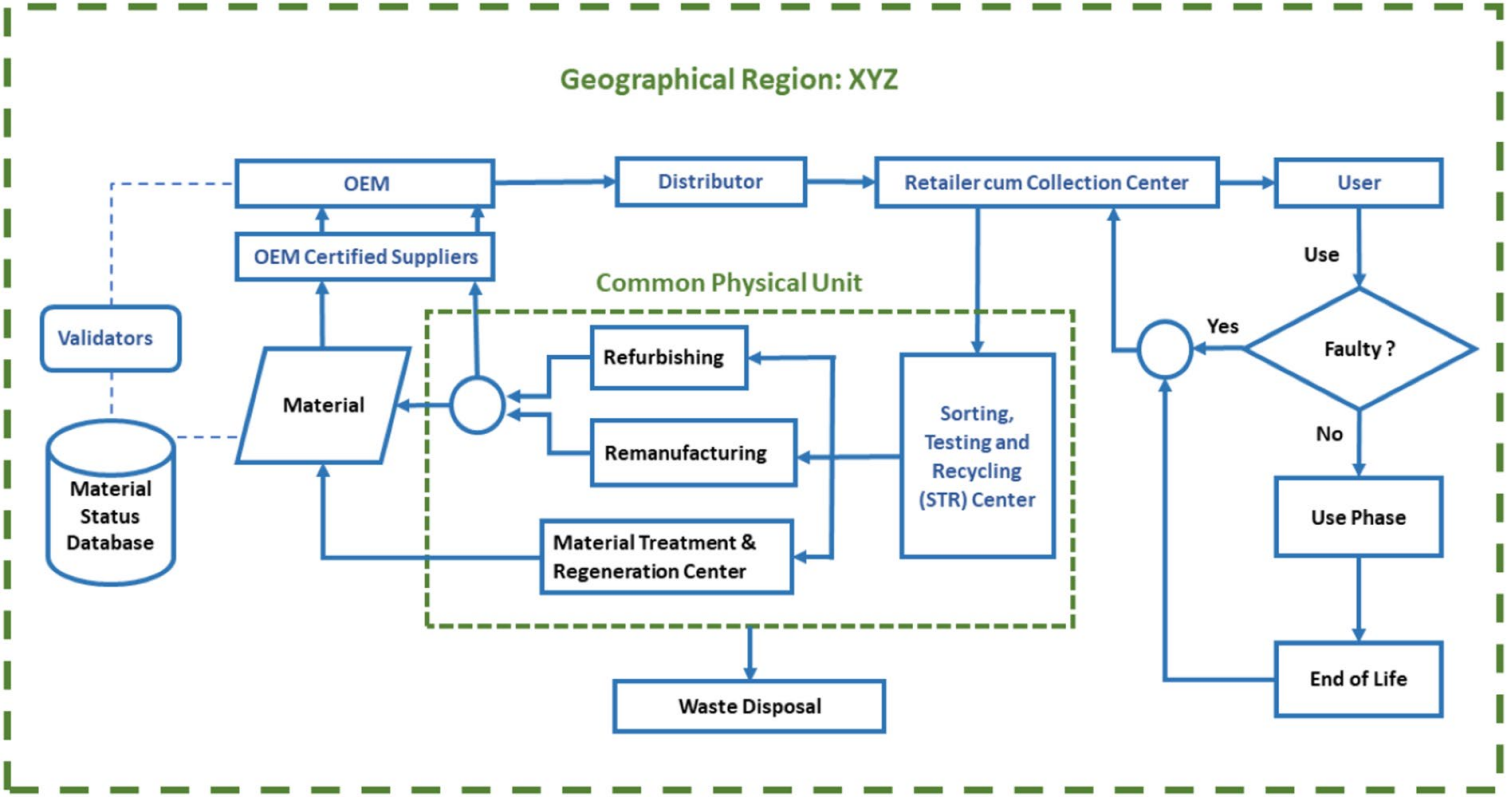
Process flow for lead acid battery circularity.

This section provides the research methodology for the proposed blockchain-enabled material level circularity architecture for hazardous and critical battery materials. Value chain actors, their roles and interrelationships are presented. The architecture has been validated with an example of a LAB value chain within a region XYZ as shown in Fig. 1. This region can be a country, a part of a country or a block of countries following similar regulations and taxation. This section also presents a mobile user interface configuration for the ease of communication among the actors.

### Blockchain actors, roles, and responsibilities

Figure 1 shows the proposed material flow architecture, and actors like OEM, distributors, retailers, recyclers, users, government authorities, validators, experts, and their interrelationships. It is proposed that each manufactured battery has a leak-proof seal and a unique QR code is laser-engraved on it. OEMs have to create a distributed ledger with all the critical and hazardous materials, their quantity and the overall battery weight to avoid gray marketing, enhance material authenticity and presents informal processing & recycling after the end-of-life. Distributors will verify the weight and seal of the batteries before distributing them to the retailers. The retailer is responsible for selling batteries to the users only after ensuring that there is no physical damage or leakage. The retailer will also work as a collection center after the end-of-life of the battery. Retailer as a collection partner was found as a convenient and most popular route as the retailer takes the old battery and replaces it with the new. Users are responsible for returning the spent or faulty batteries to the retailers. Retailers will send the returned batteries to the nearest sorting, testing, and recycling (STR) center. STR centers will inspect the used or faulty batteries and sort the batteries for refurbishing, remanufacturing, or recycling depending upon the condition of the battery. Further, the bad/damaged cells of the battery and acid are sent for scientific treatment, regeneration and disposal. The regenerated materials from the 3R (refurbishing, remanufacturing and recycling) are sent back to the OEM or its authorized suppliers and the chain is closed. As part of the blockchain network; government officials, domain experts, and third party validators monitor the batteries’ forward & backward network and validate the recovered/regenerated/disposed materials. In addition, they are authorized to validate the scientific and other genuine losses of materials along the closed loop supply chain.

### Architecture of the proposed blockchain-enabled circularity

**Figure 2:**
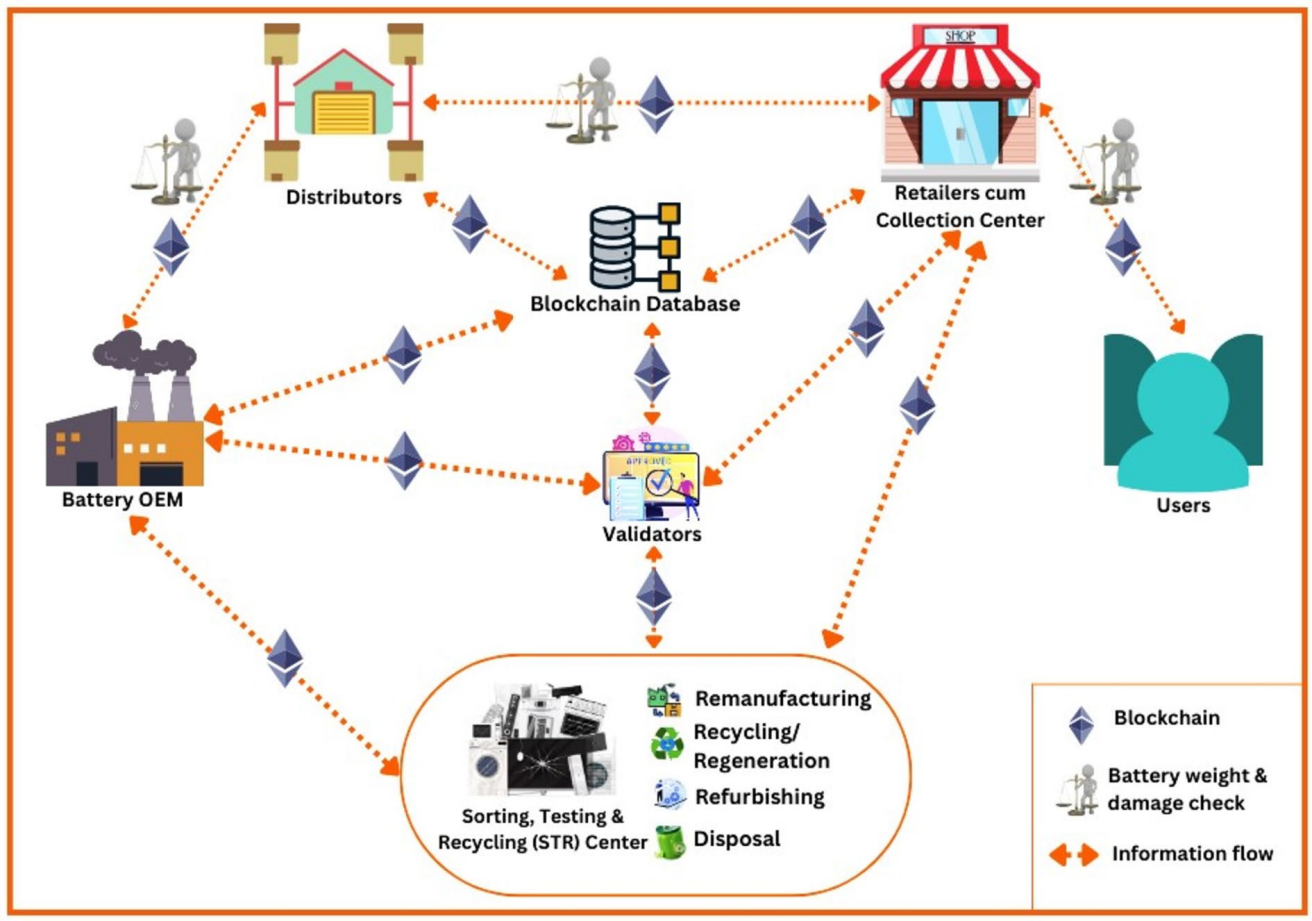
Lead acid battery blockchain architecture with information flow.

Figure 2 shows the proposed blockchain architecture and the information flow among the various actors. The information flow is continuum and all stakeholders are linked to the decentralized blockchain database. This database gets updated for every transaction by the stakeholders. A prototype system is developed using EVM (Ethereum virtual machine) based blockchain. The battery information data comes from IoT devices such as smartphones. The main actors in the whole process are the battery manufacturers (OEM), distributors, retailers, battery users/customers, government-authorized STR centers, and validators (government officials, domain experts and third party validators). As shown in Fig. 2, STR, refurbishing, remanufacturing, and regeneration centers are housed in a common physical unit for the information gathering and sharing on the blockchain. These actors have their addresses white-listed in the smart contracts to interact with blockchain. The OEM creates initial battery objects for different battery models produced and the corresponding materials (lead, tin, sulfuric acid, and the case material type), along with the assembled battery weight and the weight of materials by percentage.

The process starts when the manufacturer introduces a new battery into the supply chain by allocating a laser engraved QR code on the battery. The QR code can be generated from the app (in which the manufacturer uses a private key to sign the transaction). After QR code generation, the battery enters the blockchain as a new entry. Hence, a new battery object is created with a unique ID number linked to the OEM. The manufacturer then ships the battery to any of its distributors. After receiving the shipment, the distributor scans the QR code on the battery with the help of the distributor version of the app and signs the transaction with a private key. This results in the addition of the distributor to the respective battery. A similar process is followed for the distributor-retailer transaction.

Once the retailer has scanned the QR code and added itself on the blockchain, the battery is ready to be sold to a user. The sale is recorded by adding the user details (such as citizen card details, email addresses, and/or phone numbers) to the blockchain’s battery object with the help of the retailer’s version of the app. The sale event in the blockchain triggers an alert system that notifies the user about the battery’s health and the estimated life via email and/or SMS notification. The user can also get the address of the nearest retailer/collection center if the user moves to a different location. The user can return the battery to any white-listed retailer on the blockchain during use phase (service) or at the battery's end of life. This ends the forward supply chain of the battery.

**Figure 3:**
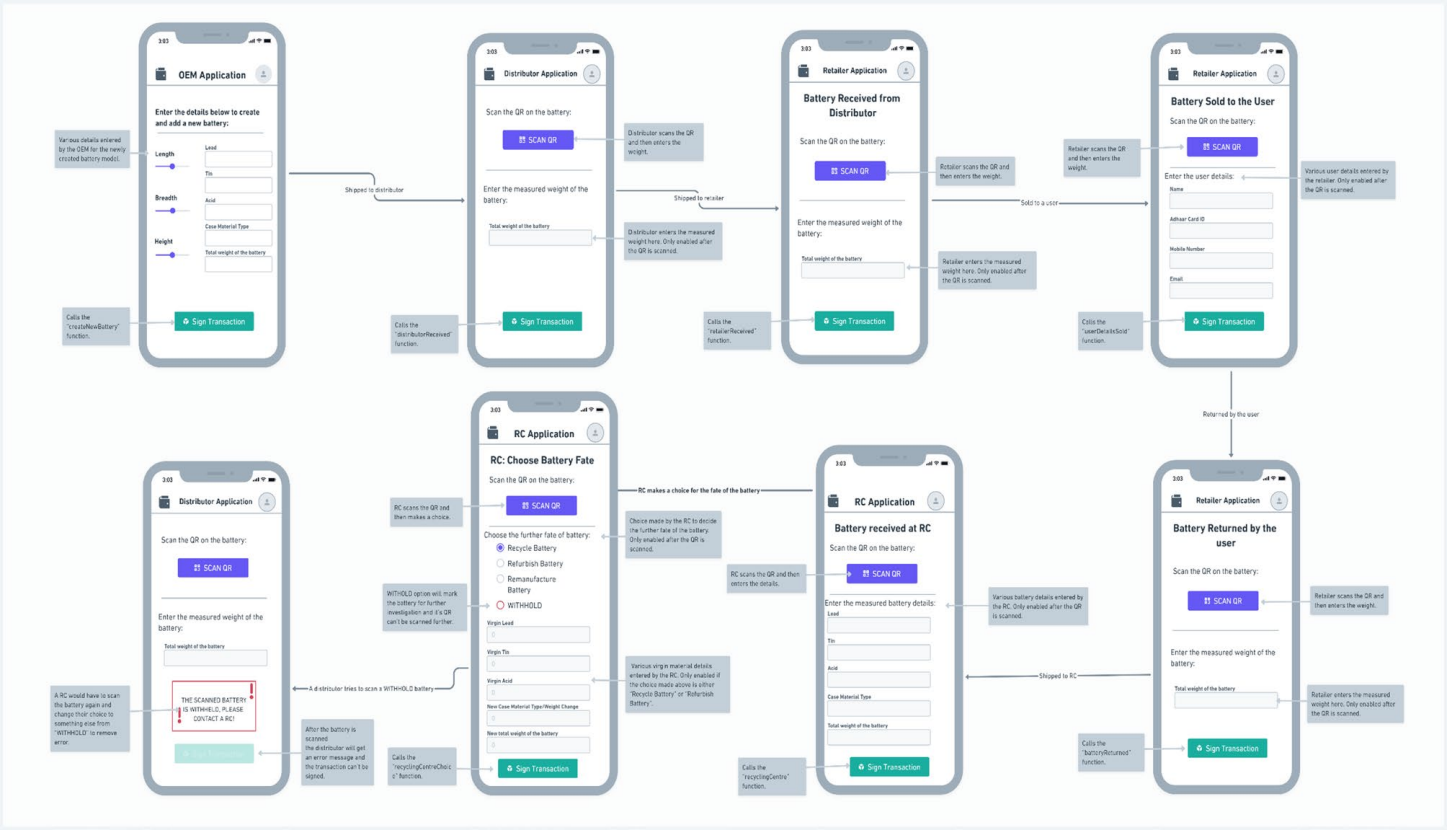
Mobile applications user interface configuration for communication among supply chain actors.

At the end of the battery life, the retailer receives the battery from the user, the retailer scans the QR code on the battery by choosing the appropriate functionality on the app, and this will update the status of the battery object on the blockchain to “returned,” and the retailer to whom the battery is returned will be added to the blockchain. The retailers will send the batteries to the nearest STR center. After inspecting, testing and analyzing the battery’s condition, the STR center will sort the batteries into three categories (3R): recycling, refurbishing, and remanufacturing. After sending the batteries to their respective categories, the recycling center will scan the QR code of the battery and thus add it to the blockchain using the respective app version and sign a transaction. Now, the battery status is updated as “completed” on the blockchain and marked as the end of the battery’s life. Refurbished/remanufactured battery will get a new “battery ID” with same old QR code. More details are provided in Sect. "Pseudo code formulation for the proposed blockchain architecture" along with the pseudo code for the ease of understanding. The information on recovered and disposed of material after the 3R treatment, will be available in the decentralized blockchain database. This database information will be helpful for validators for cross verification. It is also used by OEMs for inventory and resource management. Figure 3 shows the mobile application's user interface configuration for communication among supply chain actors. The suggested mobile dashboard for the proposed blockchain platform will be highly useful for all the actors for signing the blockchain transactions with communicable user interfaces.

This whole architecture helps in the transparent and authentic management of battery’s critical and hazardous materials such as lead, tin, acid, case material, etc. Suppose a manufacturer produces “x” batteries and each battery consumes “y” liters of acid. The number of batteries by an OEM in a geographical region ‘XYZ’ is known from the blockchain transactions, which can also be verified through sales/tax paid to the regional government authorities. Assuming a battery retain 80% of the acid at the end of the life, the government provides only the 20% virgin acid to the OEM or its authorized suppliers. The acid provided to the OEM or its authorized suppliers in the upcoming year can be computed, if the manufacturer produces “x” numbers of batteries in the next year too. This makes possible the tracing of acid and its unauthorized sale as per the law.

## Pseudo code formulation for the proposed blockchain architecture

This section demonstrates the blockchain interaction among stakeholders and gives details about the transactions used in the blockchain architecture. The pseudo code is also provided for the blockchain interaction among the actors, which can be used by future researchers to generate their own codes for different cases.

The cycle is started when the OEM scans a newly manufactured battery via an IoT device, which detects the battery model using its pre-defined dimensions and shape.
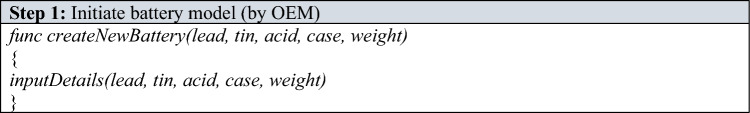


Once the battery model is detected, a function call is made to the smart contract, thus marks the battery object as “active” (or status code 1) and generates a QR code.
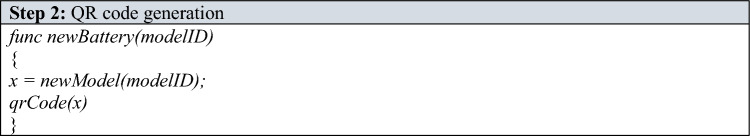


Battery model ID passes an argument to this function as the IoT detects it. Overloaded variations (with different parameters) of this function exists and are referred to in the “refurbBattery()” and the “remanuBattery()” functions below.

Now the battery is in the active state (when the newModel() is called initially, the activeState field is set as “1” of the created new battery object). The generated QR code is physically “laser engraved” on the battery. Once the battery is shipped by the OEM and received by the appropriate distributor, the distributor scans the QR code on the battery via a smart phone and calls a function on the smart contract by initiating a transaction using private key. This marks the battery object as received by the particular distributor. Next, the distributor measures the weight of the received battery and enters the measured weight as an argument while calling the function. Only authorized distributors will have white-listed addresses to call this function.
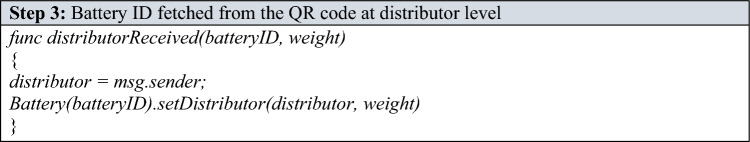


The battery object has “public” getter methods. Hence, anyone can view relevant information (manufacturer, distributor, date of manufacturing, date of receiving by distributor, etc.) by simply interacting with the smart contract. Once the distributor ships the battery to the retailer, the retailer follows a procedure similar to the distributor’s procedure and calls a function associating the retailer with the particular battery. Only authorized retailers will have white-listed addresses to call this function.
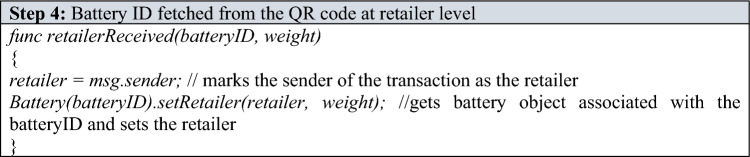


The retailer also measures the weight of the received battery and enters the measured weight as an argument while calling the function. Once the battery is ready to be sold to a user, the retailer scans the QR code again and enters the user's details to whom the battery is being sold as arguments to the function.
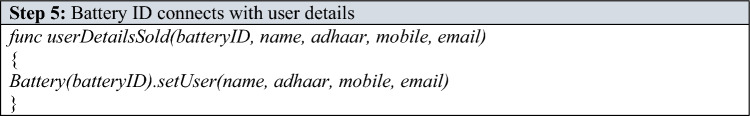


The adhaar is cryptographically hashed before storage and hence is not available in plain text form in the blockchain but is verifiable and thus protects the user’s privacy.

The battery is now in the use phase. The customer/user will be notified about the battery health status (based on approximations and standard decay calculations) via SMS and email services, reminding them of an approximate return date to the retailer. If the battery has a fault, the user can return the battery to the retailer.

When the user returns the battery to the retailer, the battery’s weight is measured. After scanning the QR code associated with the battery, a function call is made to the smart contract(s) with the measured weight as an argument to the call made.
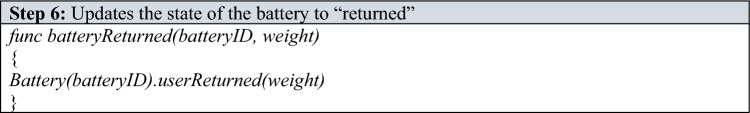


The retailer delivers the battery to the nearest government-authorized recycling center, a trusted entity in the proposed architecture. The recycling center performs an in-depth health inspection of the received battery. It reports the lead, tin, and sulfuric acid content, along with the weight percentage for each component mentioned (inclusive of the case of the battery) and the total weight of the final assembled battery via calling a function on the smart contract by scanning the QR code and passes all the information as arguments to the function call.
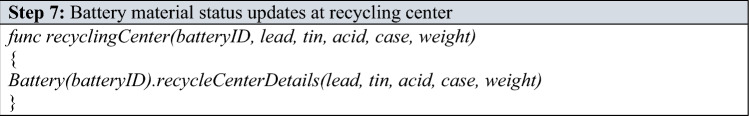


Based on the physical condition, battery health, and condition of the battery components, the recycling center decides to sort the battery and allocate to one of the categories—Refurbishing, Remanufacturing or Recycling.
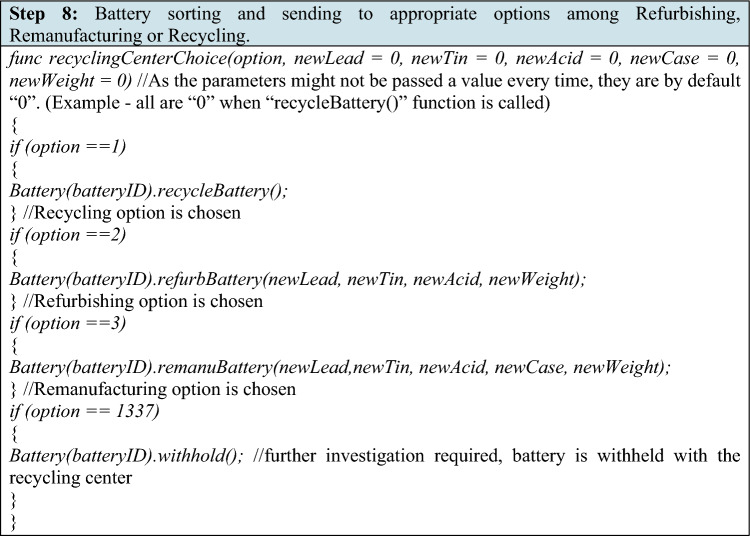


The details for each of the options are as follows:

Recycling—If the recycling center decides to recycle the battery, all the materials are recovered by the recycling center. The “recycleBattery()” function is called, which marks the activeState field of that battery object as “02” (recycled and closed) and scanning the QR code printed on the physical case won’t initiate any function call even though all of the data associated with the battery object corresponding to the batteryID remains on the blockchain.
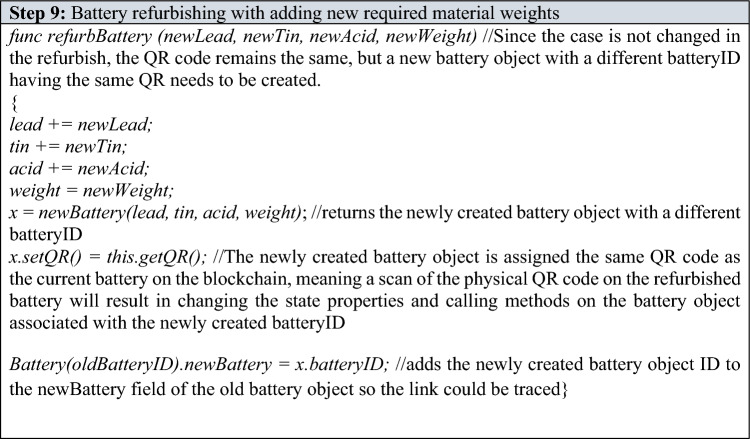


Refurbishing and Remanufacturing—If the recycling center decides that the battery should be remanufactured/refurbished, and it requires a certain amount of virgin materials to be added, the recycling center will measure and add the required virgin materials to the battery and then call the appropriate value to the “option” parameter of the “recyclingCenterChoice()” function thus executing either “refurbBattery()” or “remanuBattery()” function. The newly added virgin lead, tin and acid content along with the new total weight of the battery are passed as arguments if calling “refurbBattery()”. Similarly, newly added virgin lead, tin, acid and the new case type weight (if the case is being changed) along with the new total weight are passed as arguments if calling “remanuBattery()”. If case is not being changed and remanufacturing is chosen, “0” can be passed as a value to the “newCase” parameter in the “remanuBattery” function.
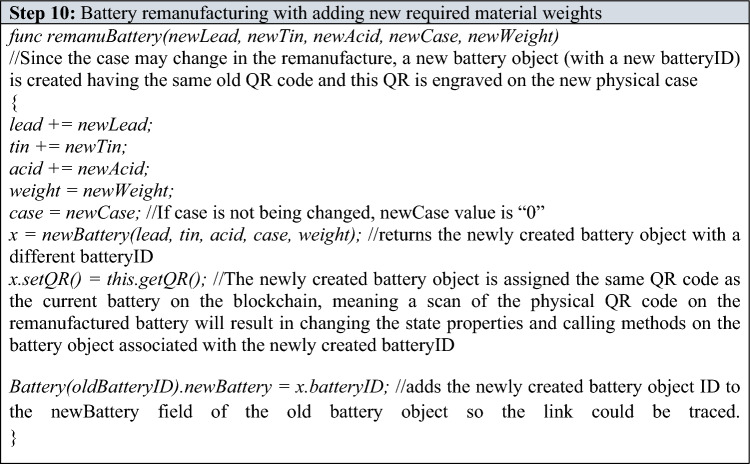


The recycling center sends the recovered materials (in the case of recycling) and the battery (in the case of refurbishing and remanufacturing) back in the loop, where its new cycle will begin with a new batteryID as depicted in the functions above. In case of any discrepancies in the content and amounts of various substances, which are measured and calculated by the recycling center, the recycling center may withhold the battery physically and demands further investigation on that particular battery. In such a case, the recycling center must pass “1337” as a the value to the “option” parameter in the function “recyclingCenterChoice()” which will, in turn, call the “withhold()” function on the battery object and will change the activeState value of that battery object to “1337” and thus marking the battery as “investigation and attention required by the authorities.”

## Technological and managerial implications

Recently, Singh et al.^40^ identified many technological, organizational, environmental, and market driven barriers to the adoption of blockchain in construction industry; and found technology immaturity and accessibility as the critical barriers. Data tapping and verification is a big challenge for the successful deployment and acceptance of the blockchain-based solutions in the industry. Manually entered data needs to be verified authentically. Third party verifiers are required to audit the manually entered data to the blockchain. Internet of Things (IoT) based solution is another way to capture and record the live data on the blockchain. Khalil et al.^41^ proposed expansion in non-fungible tokens (NFTs) for IoT asset representation to provide device and user identification and authentic functionality. Recently, oracle-based blockchain has started incorporating oracles, which are external services or agents that provide real-world data to smart contracts running on the blockchain. These oracles act as bridges between the blockchain and external sources of information, enabling the blockchain to interact with data that exists outside of its network. However, an easy to adopt yet transparent data tapping mechanism is still a challenge for the blockchain-based industry solutions. The blockchain-based solutions in industrial applications are in nascent stage, the technology is growing but had not yet matured.

Interoperability is another issue for the blockchain technology deployment as different stakeholders across the supply chain may be using different platforms and communication protocols. There is still a lack of interoperability among different blockchain platforms like Ethereum, Hyperledger, etc. Scalability issues associated with decentralized systems like blockchain, which store a copy of transactions at all nodes, makes the transactions slower. Modular cross-technological platform interfaces, and mergeable & scalable blockchains are some of the solutions to improve integrability and scalability of blockchains^42^.

Traditional barriers like reluctance to adopt new systems, organizational culture, market uncertainty, etc. also act as barriers to blockchain adoption. Unawareness and resistance to blockchain technology among customers is a significant barrier^40^. A carrot and stick policy can be used to force the OEMs to adopt material circularity through regulation and motivate external stakeholders through incentives and rewards.

Initial investment for infrastructure development at the supply chain stakeholders; maintenance and support costs; operations related costs like energy consumption, and transaction gas fees; training and employee onboarding; etc. are some of the challenges faced by the managers during the adoption of blockchain technology for LAB circularity.

Data privacy and security must be handled properly along the supply chain. The proposed framework uses Ethereum-based blockchain, which supports Aztec Protocol to improve privacy. Smart contracts-based access controls and data masking is also used to limit the exposure of confidential and sensitive data. These methods jointly ensure that sensitive information remains confidential along supply chain. However, privacy of sensitive information can be further protected through advanced cryptographic techniques like zero-knowledge proofs^43–45^ and homomorphic encryption. Privacy-preserving protocols such as confidential transactions and “layer 2” solutions, and off-chain storage using decentralized networks like Inter Planetary File System (IPFS) have been developed to improve privacy and security. Security issues can also be achieved by using properly designed NFT architecture^41^.

## Conclusions and outlook

This paper proposes a blockchain-enabled circularity architeture for the LABs to improve the traceability, authenticity and transparency of the battery and its critical and hazardous materials. The proposed architecture helps the original equipment manufacturers in tracing the products and the material consumption, provides visible tool for supporting SDG 12 (responsible consumption and production) and supports extended producer responsibility. Another significant contribution of the proposed architecture is improved authenticity for the safe treatment and scientific disposal of the hazardous materials particularly acid, which is not economical to dispose of scientifically.

- The proposed architecture defines accountability and ensures the participation of each actor throughout the life cycle and disposal of the battery.
- A product can be traced throughout the supply chain, which helps in preventing non-professional disposal and/or treatment of critical and hazardous materials.
- The proposed architecture is user-friendly to the stakeholders in the value chain.
- Ensures the extended producer’s responsibility for recycling their own products and keeping the check on virgin and secondary (recycled) material used in the manufacturing of the product. Material level accounting helps in fulfilling SDG goal 12, i.e., responsible consumption and production.
- Mobile App for the blockchain makes the system more interactive and prompt due to simple user interface.
- The database provides the information of net material required to produce a unit product and keeps the information on recovered material after recycling, which helps in managing resources.

The proposed circularity architecture is generic but the blockchain coding is for LAB circularity. However, the codes can be easily rewritten for any other product. It is expected that the architecture will be useful for the regulatory authorities to keep a tab on illegal disposal of hazardous substances. This architecture can be extended further by incorporating the concept of blockchain interoperability for multiple products and multiple OEMs. This paper can be further developed to find the economic viability of the proposed approach in terms of hardware and software costs required for the set-up and integration of the blockchain technology at various levels—OEM, distributers, retailers, users, STR centers, validators, etc.; maintenance and support costs; operations related costs like energy consumption, and transaction gas fees; training and employee onboarding, etc. Artificial Intelligence techniques^42^ and IoT^41^ have been used to improve effectiveness and authenticity of blockchain technology, respectively. These areas should be further explored to enhance the blockchain effectiveness and adoptability.

## Data Availability

All data generated or analysed during this study are included in this published article.
